# Drill-and-prechop technique: modification of the drill-and-crack technique for mature cataracts

**DOI:** 10.1186/s12886-022-02671-w

**Published:** 2022-11-22

**Authors:** Mao Xu, Yongjun Qi, Yongde Weng, Yang Yang, Jianhua Deng, Wanjun Liu, Ting Mo, Xiangxiang Ye

**Affiliations:** 1 The First School of Clinical Medicine, Guangzhou University of Chinese Medicine, Guangzhou, China; 2 Guangdong Hospital of Traditional Chinese Medicine, Zhuhai, China; 3 TCM-Integrated Hospital of Southern Medical University, Guangzhou, China

**Keywords:** Chop, Microincision, Phacoemulsification, Prechop, Hard nucleus, Teaching phaco

## Abstract

**Background:**

There are some techniques for disassembly of hard nuclear. It is challenging in hard cataract surgery through microincision. The classic chop or prechop techniques often do not succeed,resulting in incomplete nuclear segmentation. The authors describe a new chop technique for removing hard nucleus cataracts in coaxial microincisional cataract surgery.

**Methods:**

We create a deep hole (drill) in the central nucleus with the phaco tip and divide the nucleus (prechop) with the Nagahara chopper and the modified capsulorhexis forceps inside the hole. The chopper and the modified capsulorhexis forceps are spread apart laterally after they approach at the center of the nucleus, to create a complete fracture across the entire nucleus. Since January 2022, we have completed 27 eyes of 25 patients with hard nucleus cataract using this technique.

**Results:**

Complete segmentation of the hard nuclear into two hemispheres was implemented with this drill and prechop technique in all cases. The effective phaco time and ultrasound energy decreased. No intraoperative complication such as iris injury, anterior capsule tears, zonulysis, or posterior capsule rupture with vitreous loss occurred during surgery.

**Conclusions:**

This technique simplifies the previous prechop techniques especially for hard nucleus in microincisional cataract surgery. The technique is efficient, safe and simple.

**Supplementary Information:**

The online version contains supplementary material available at 10.1186/s12886-022-02671-w.

## Background

Phacoemulsification of mature cataracts is more widely performed than extracapsular cataract extraction even in the Chinese rural population. However,it has always been a challenge to perform coaxial microincision phacoemulsification of hard cataracts. The absence of a protective epinuclear layer, the paucity of cortex, the fragility of the capsule, and the laxity of the zonules increase the risk of injury to the supportive structures of the lens during surgery [1]. Many techniques for hard cataracts have been described: step-by-step chop in situ [2], crater-and-chop technique [3], drill and chop [4], multilevel chop technique [5], terminal chop [1],consecutive drilling combined with phaco chop [6], and rotary chop [7]. To minimize the ultrasound energy in phacoemulsification, many prechop techniques have been described [6, 8–12]. However, they require additional special instruments or the additional expensive femtosecond-laser platform. Some manual prechop techniques could not easily complete segmentation of the dense nuclear into two hemispheres.

We introduce a modification of the drill-and-crack technique introduced by Hwang HS et al. [13] that can be used safely and effectively to remove hard nucleus. With our technique, the very hard nucleus can be split through the posterior plate without stress to zonular fibres. We propose that this alternative technique is smooth, safe, and effective, with a short learning curve and excellent early postoperative visual outcomes.

## Methods

**Fig. 1:**
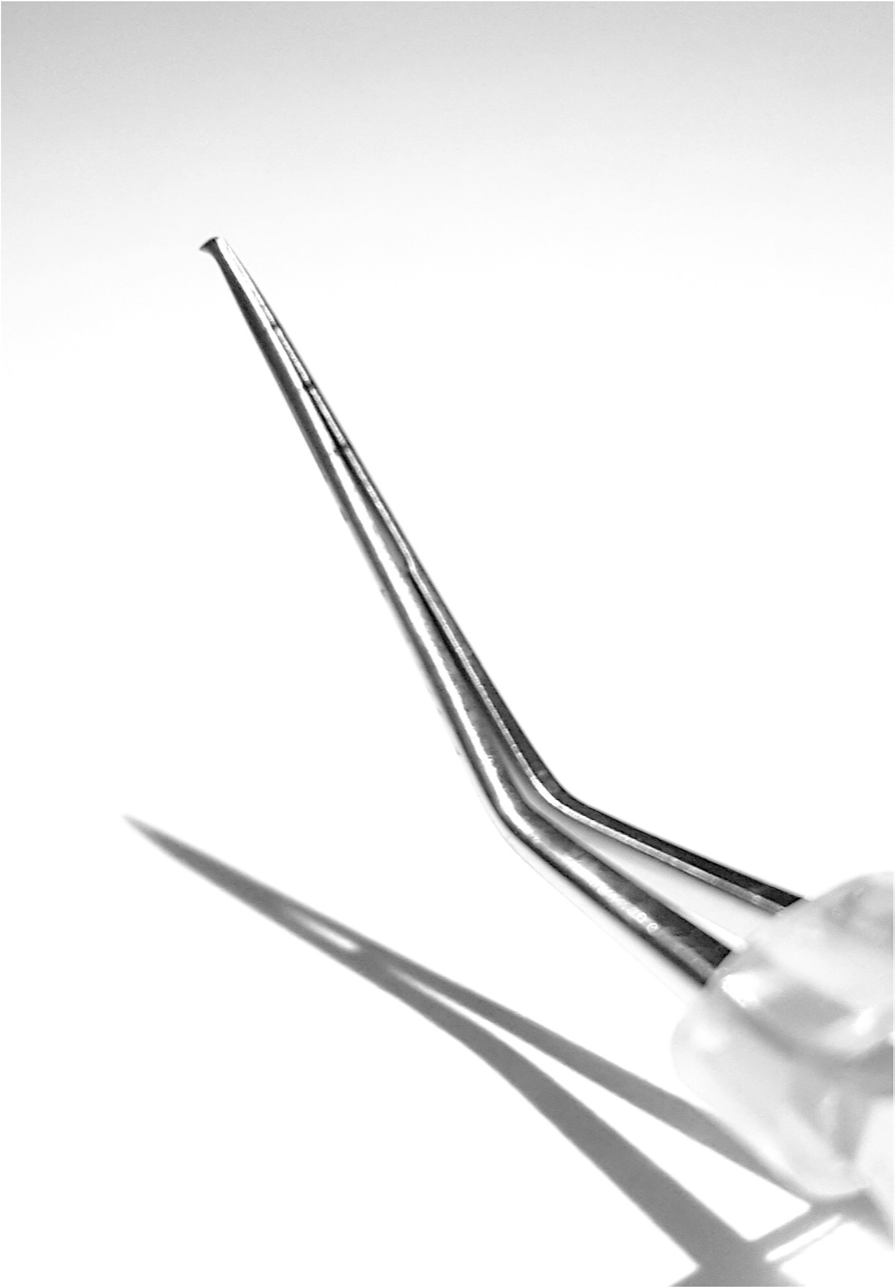
The sharp triangular forceps head of the modified capsulorhexis forceps

No additional equipment is required for this technique compared with traditional phaco beyond a capsulorhexis forceps (Mingren, Suzhou, China, patent no: ZL201520078867.9, Fig.1). It has a sharp triangular forceps head looking like the bow of a warship when it closes.

Our video example uses a megaTRON S4 System (Geuder, Heidelberg, Germany) with the following parameters: longitudinal phaco at 60% power in continuous mode, 90 cm bottle height, 350 mmHg of vacuum, and 30 cc/min of aspiration flow.

### Surgical technique

**Fig. 2:**
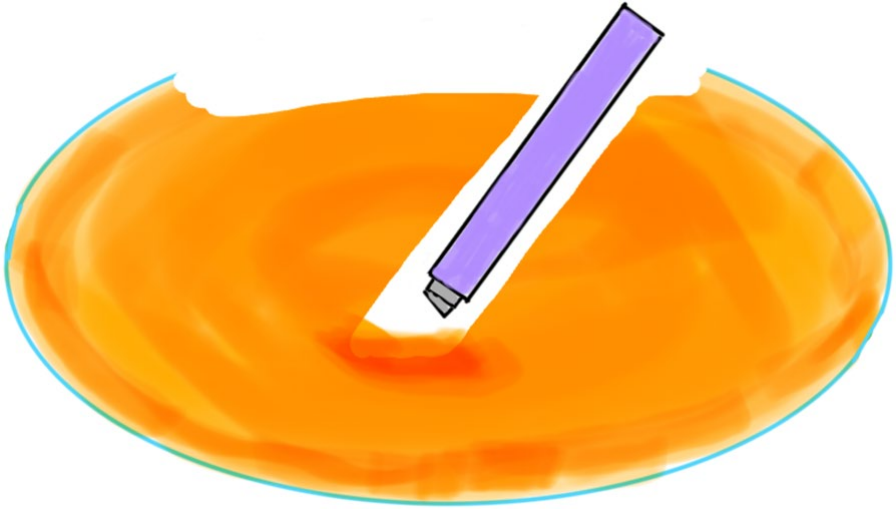
With the phaco tip bevel down, facing the nucleus, a deep, narrow hole is made by embedding the phaco tip near the geometric centre of the endonucleus

**Fig. 3:**
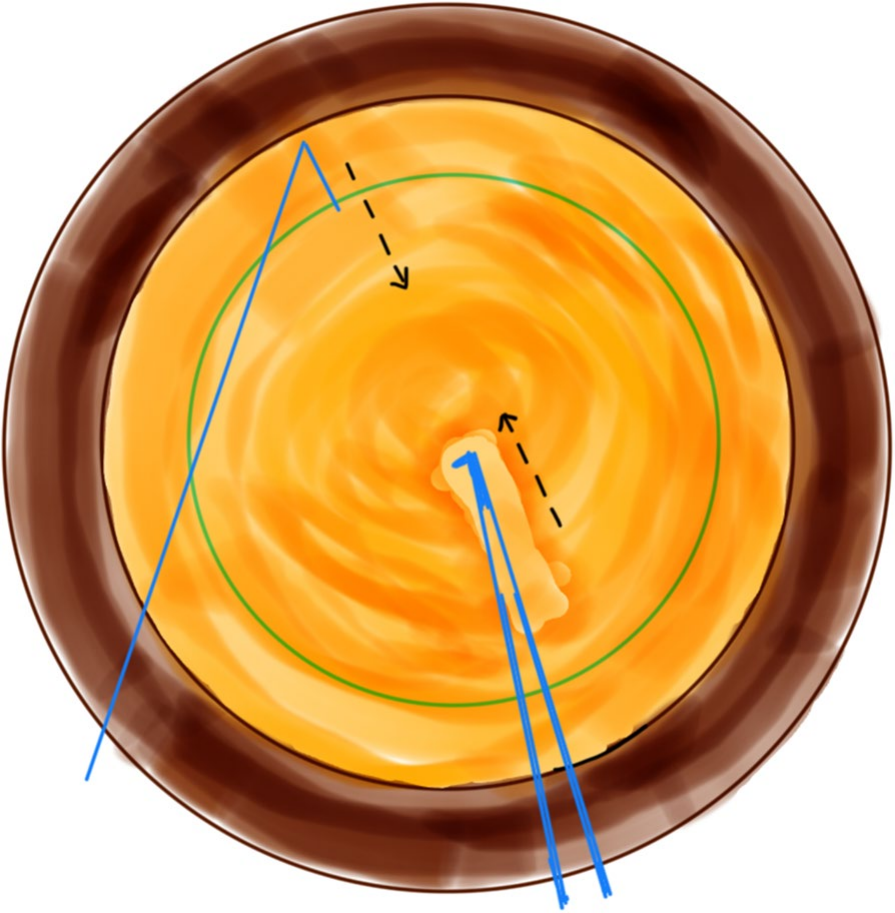
The chopper is slid under the capsule and rotated resting at 5 o’clock in the bag, maintaining the chopper tip toward the hole. The capsulorhexis forceps was inserted into the hole

**Fig. 4:**
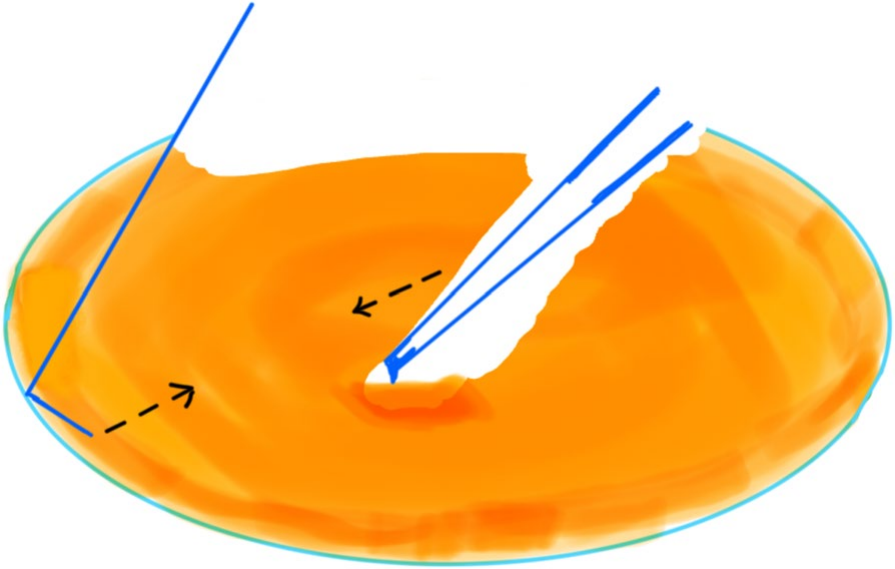
The chopper is pulled obliquely up toward the centre along the lens fibre, while the capsulorhexis forceps is pushed slightly obliquely down to provide a counterforce

**Fig. 5:**
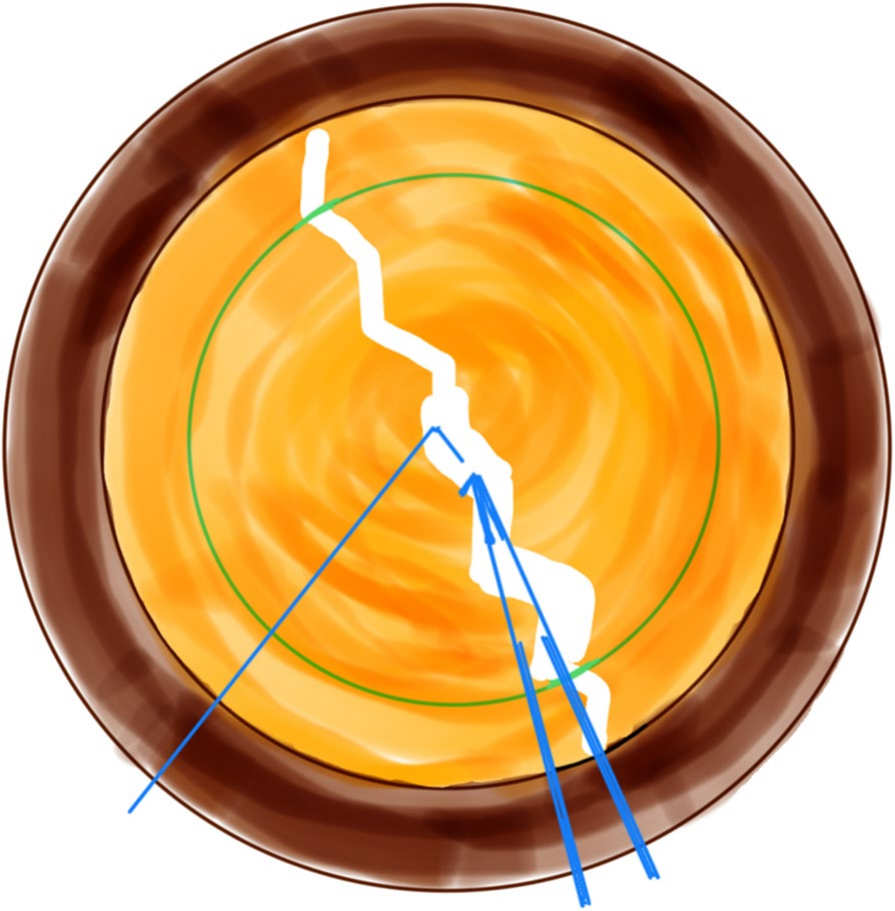
The nucleus will be split completely into two hemispheres

**Fig. 6:**
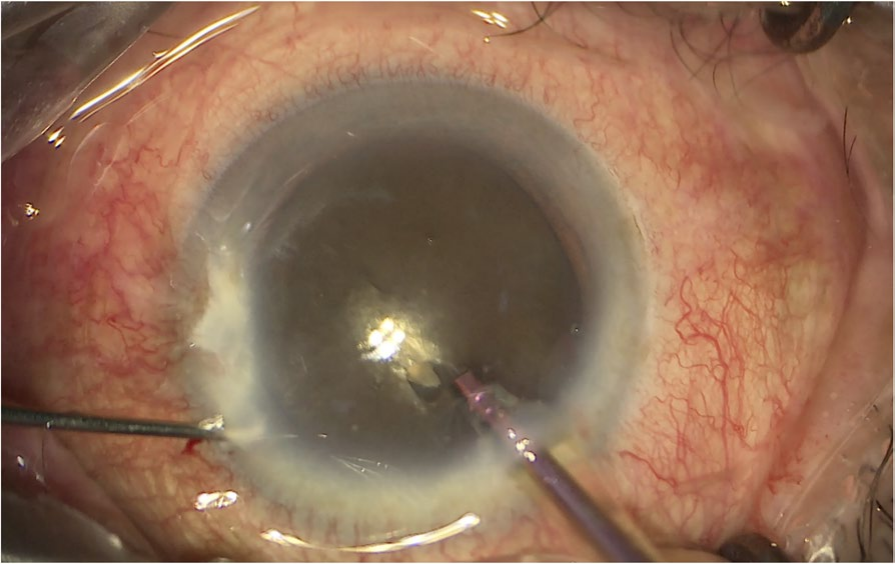
Create a deep hole (drill) near the geometric centre of the endonucleus

**Fig. 7:**
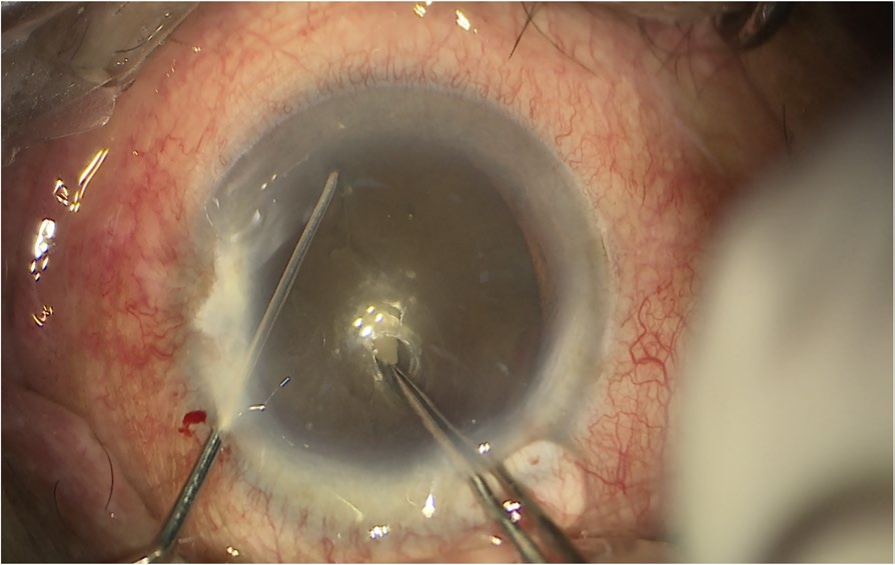
Divide the nucleus (prechop) with the Nagahara chopper and the modified capsulorhexis forceps inside the hole

**Fig. 8:**
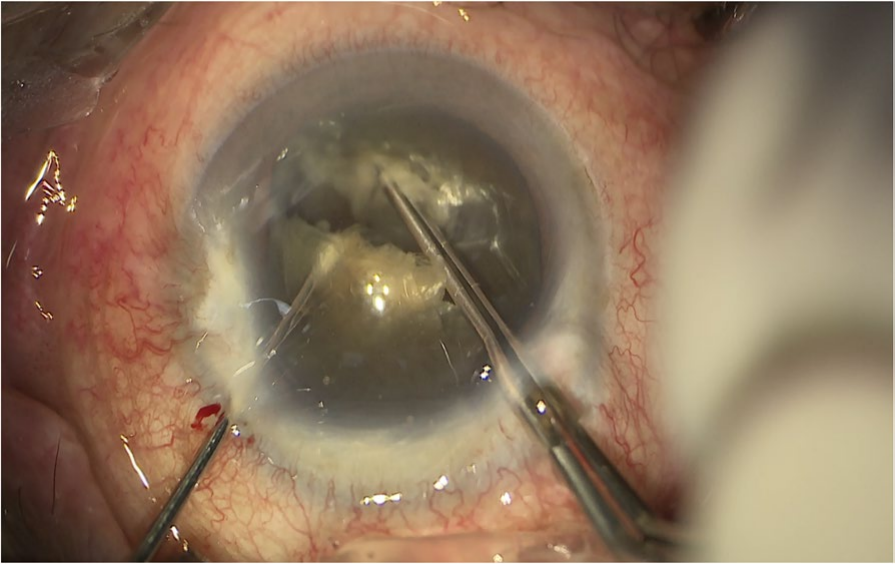
The Nagahara chopper and the modified capsulorhexis forceps are gently separated laterally so the nucleus will be split completely into two hemispheres

All coaxial microincisional cataract surgery (coaxial MICS) is performed under topical anaesthesia through a clear corneal incision with a side port. The size of main incision is 2.2 mm. After a continuous curvilinear capsulorhexis is made, the exposed phaco tip is extended to about 1.5–2.0 mm and then inserted into the anterior chamber with the bevel down. The superficial cortex is removed. With the phaco tip bevel down facing the nucleus, a deep, narrow hole is made by embedding the phaco tip from the mid periphery area next to the capsulotomy edge (Fig.2). The angle of drilling is approximately 60°,and the depth is approximately two-thirds depth of the anteroposterior lens thickness, which can be measured by placing the phaco tip with a known diameter. When the entire exposed phaco tip has been embedded in the nucleus, it is removed and sodium hyaluronate is injected into the anterior chamber. The long Nagahara chopper is introduced into the anterior chamber through the paracentesis. The nucleus is gently engaged by the capsulorhexis forceps at the anterior pole, followed by a slight pull toward the main incision. Simultaneously, the chopper is slid under the capsule and rotated so the blade is perpendicular to the equator of the nucleus, resting at 5 o’clock in the bag, maintaining the chopper tip toward the hole. By this time, the capsulorhexis forceps has been repositioned and inserted into the hole. The Nagahara chopper and the capsulorhexis forceps are kept in apposition at the same radial meridian. The chopper is pulled obliquely up toward the centre along the lens fibre, while the capsulorhexis forceps is pushed slightly obliquely down to provide a counterforce (Fig. 3–4). Once they meet, the capsulorhexis forceps is unfolded slightly, repeat the opening procedures to complete the division over the entire depth and length of the nucleus until the posterior capsule is reached,then two instruments are gently separated laterally so the nucleus will be split completely into two hemispheres (Fig. 5). The hemispheres are rotated by the chopper and divided into quadrants. Each fragment is then aspirated and emulsified within the capsular bag. The main operation steps are shown in the operation screenshot (Figs. 6, 7 and 8).

### Patients

The drill and prechop technique has been adopted in 27 eyes of 25 patients (average age 77.3 ± 3.6 years) with cataract harder than grade 4 according to Emery-Little classification in the past 6 months. There were 12 male patients and 13 female patients. The preoperative BCVA was less than 0.1 in 10 eyes (37%), 0.1–0.3 in 11 eyes (41%), and 0.3–0.4 in 7 eyes (22%).All patients signed an informed consent for participation in this study. All patients were treated in accordance with compliance guidelines outlined by the Declaration of Helsinki.

## Results

Since January 2022, we have completed 27 eyes of 25 patients using this technique. In all cases,the division over the entire depth and length of the nucleus including the posterior plate is completed. The postoperative BCVA was less than 0.3 in 2 eyes (7%), 0.3–0.5 in 5 eyes (18.5%), and 0.5–0.8 in 20 eyes (74.5%).The postoperative visual acuity of each patient has been improved. The average intraoperative effective phaco time displayed on the megaTRON S4 System was 9 s using this technique. When using phaco-chop before, the average intraoperative effective phaco time was 15 seconds. No intraoperative complication such as iris injury, anterior capsule tears, zonulysis, or posterior capsule rupture with vitreous loss occurred during surgery.

## Discussion

Complete splitting of the nucleus into two hemispheres is an essential step in accomplishing uneventful coaxial microincision phacoemulsification of mature cataracts. Endothelial cell loss was initially increased in MICS, especially in eyes with increased nuclear density because of increased cumulative dissipated energy, aspiration time, and volume of balanced salt solution used [14]. Some advanced phacoemulsification techniques may also decrease energy use. However, the techniques including various prechop techniques quite often does not succeed, resulting in incomplete nuclear segmentation and intact posterior plate, or require additional special prechop instruments.

The consecutive drilling combined with phaco chop technique introduced by Chen D et al. [6] to remove hard cataracts in MICS involves 3–4 holes consecutively drilled into the endonucleus. The nucleus is deeply impaled with the last drilling and firmly engaged with high vacuum, and then chopped with chopper centripetally from the lens equator. The chopper and phaco tip are spread apart laterally after they approach at the centre of the nucleus. It combines the advantages of phaco-chop and phaco-drill [15]. Rotary chop introduced by Ifantides C et al. [7] created two or more partial thickness pilot holes around the periphery of the nucleus by the phaco tip. Then, tackle the nucleus with the chopper and phaco tip in two holes in a meridian. At least two holes need to be created in the technique. Moreover, drilling holes with high ultrasound energy may increase the risk of complications such as corneal burn and corneal endothelial damage in mature cataracts in MICS.

The drill-and-crack technique, introduced by Hwang HS et al. [13] involves a deep hole in the central nucleus with the phaco tip and divides the nucleus with the prechopper inside the hole. Different from the consecutive drilling combined with the phaco chop technique [6] and rotary chop [7], it only drills one deep, narrow hole near the geometric centre of the endonucleus which could decrease the ultrasound energy. It offers easy and effective nuclear disassembly in patients with a hard nucleus cataract. However, it needs an Akahoshi prechopper [8, 10]. In microincisional cataract surgery, especially in 1.8 or 2.2 mm incision surgery, it is difficult to fully deploy the prechopper near the main incision and completely separate the posterior plate.

We adopted the drill-and-prechop idea from the drill-and-crack technique [13] and the cystotome-assisted prechop technique [16]. After digging a narrow, deep hole near the centre of the endonucleus, we used the Nagaharra chopper and the modified capsulorhexis forceps to split the nucleus. It can be considered a prechop technique. No phaco energy is wasted in the prechop procedure. We made a new capsulorhexis forceps which can perform the capsulorhexis and prechop. The modified capsulorhexis forceps (Fig.1) has a sharp triangular forceps head similar to a bow of a warship when it closes. Because of this feature, the nucleus is easily split into two unconnected hemispheres.

In the cystotome-assisted prechop technique [16], after the capsulorhexis, the surgeon-bent cystotome is inserted into the lens while the Nagahara chopper is set around the lens equator. The cystotome and the chopper are then brought together in the centre to create a bisecting crack in the nucleus, dividing it into two hemispheres. The nucleus of grade 3 according to Emery-Little classification is easy to split, but when splitting the grade 4 or 5 nucleus, the nucleus may rotate or cannot completely split the posterior plate. We replace the cystotome with our modified capsulorhexis forceps to do the prechop. When we firstly dig a hole in the endonucleus, the hardest part of the nucleus was wrecked. Then the counterforce of the chopper and the capsulorhexis forceps can totally split the nucleus.

We performed the bisection without hydrodissection. Xu Chen et al. [16] made the point that after hydrodissection, the endonucleus may be rotatable, which makes holding and bisecting it difficult or impossible in the manual prechop technique. The surgeon can also choose to perform additional hydrodissection after the nucleus bisection procedure. Then, the hemispheres are rotated by the chopper and divided into quadrants.

Like other prechop technique, no phaco energy is wasted in drill and prechop procedure. It is not necessary to build the occlusion in the endonucleus with precise pedal control and a high vacuum, which eliminates the difficulty in the chopping procedure and reduce use of the ultrasound energy.

## Conclusions

In summary, this technique could simplify the previous prechop techniques especially in MICS. It does not require additional specialized prechop instruments. It reduces the phaco time and the damage to corneal endothelial cells. It is easy to learn. It is an efficient, safe, simple, and swift procedure for full-thickness nuclear segmentation,giving consistent results,especially in hard mature cataracts. It will be helpful to surgeons.

## Supplementary Information

**Additional file 1.**

## Data Availability

Data are available upon request due to concerns about potential breach of confidentiality. The dataset is only available upon request to qualified researchers. Please contact the first author, Dr. Mao Xu, for access to the data set.

## Abbreviations

MICS: Microincisional cataract surgery

## References

[1] Prasad R, Badhani A, Dogra GB (2017). Terminal chop: new technique for full thickness nuclear segmentation in mature hard cataract. Indian J Ophthalmol.

[2] Vasavada A, Singh R (1998). Step-by-step chop in situ and separation of very dense cataracts. J Cataract Refract Surg.

[3] Vanathi M, Vajpayee RB, Tandon R (2001). Crater-and-chop technique for phacoemulsification of hard cataracts. J Cataract Refract Surg.

[4] Kim DY, Jang JH (2012). Drill and chop: modified vertical chop technique for hard cataract. Ophthalmic Surg Lasers Imaging.

[5] Vasavada AR, Raj SM (2011). Multilevel chop technique. J Cataract Refract Surg.

[6] Chen D, Tang Q, Yu F, et al. Consecutive drilling combined with phaco chop for full thickness segmentation of very hard nucleus in coaxial microincisional cataract surgery. BMC Ophthalmol. 2019;19(1). 10.1186/s12886-019-1033-1 PMC6335814 30651088

[7] Ifantides C, Sieck EG, Christopher KL (2020). Rotary chop: a new technique for teaching chop and tackling mature cataracts. Ophthalmol Ther.

[8] Akahoshi T (1998). Phaco prechop: manual nucleofracture prior to phacoemulsification. Operative Tech Cataract Refract Surg.

[9] Henriques JS, Alio JL, Akahoshi T (2009). Prechopping surgical techniques. Tech Ophthalmol.

[10] Akahoshi T, Yuan J-Q, Lim ASM (2001). Phaco prechop: mechanical nucleofracture prior to phacoemulsification. The frontier of ophthalmology in the 21st century.

[11] Bhatti SS (2009). Description of surgical technique: the Bhatti modification for small-incision cataract surgery of the Akahoshi prechop technique. Indian J Ophthalmol.

[12] Berger A, Contin IN, Nicoletti G (2012). Middle prechop: fracturing the middle portion of the nucleus. J Cataract Refract Surg.

[13] Hwang HS, Kim EC, Kim MS (2010). Drill-and-crack technique for nuclear disassembly of hard nucleus. J Cataract Refract Surg.

[14] Mahdy MAS, Eid MZ, Mohammed MA-B (2012). Relationship between endothelial cell loss and microcoaxial phacoemulsification parameters in noncomplicated cataract surgery. Clin Ophthalmol.

[15] Joo C-K, Kim YH (1997). Phacoemulsification with a bevel-down phaco tip: phaco-drill. J Cataract Refract Surg.

[16] Chen X, Liu B, Xiao Y (2015). Cystotome-assisted prechop technique. J Cataract Refract Surg.

[17] Moshirfar M, Churgin DS, Hsu M (2011). Femtosecond laser-assisted cataract surgery: a current review. Middle East Afr J Ophthalmol.

